# Cancer cell-selective, clathrin-mediated endocytosis of aptamer decorated nanoparticles

**DOI:** 10.18632/oncotarget.24772

**Published:** 2018-04-20

**Authors:** Shira Engelberg, Julia Modrejewski, Johanna G. Walter, Yoav D. Livney, Yehuda G. Assaraf

**Affiliations:** ^1^ The Laboratory of Food Physical Chemistry and Biopolymeric Delivery Systems, Department of Biotechnology and Food Engineering, Technion, Israel Institute of Technology, Haifa, Israel; ^2^ Institute for Technical Chemistry, Leibniz University Hannover, Lower Saxony, Germany; ^3^ The Fred Wyszkowski Cancer Research Laboratory, Department of Biology, Technion-Israel Institute of Technology, Haifa, Israel

**Keywords:** lung cancer, targeted delivery, aptamers, clathrin-mediated endocytosis, multidrug resistance

## Abstract

Lung cancer is the leading cause of cancer mortality worldwide, resulting in 88% deaths of all diagnosed patients. Hence, novel therapeutic modalities are urgently needed. Single-stranded oligonucleotide-based aptamers (APTs) are excellent ligands for tumor cell targeting. However, the molecular mechanisms underlying their internalization into living cells have been poorly studied. Towards the application of APTs for active drug targeting to cancer cells, we herein studied the mechanism underlying S15-APT internalization into human non-small cell lung cancer A549 cells. We thus delineated the mode of entry of a model nanomedical system based on quantum dots (QDs) decorated with S15-APTs as a selective targeting moiety for uptake by A549 cells. These APT-decorated QDs displayed selective binding to, and internalization by target A549 cells, but not by normal human bronchial epithelial BEAS2B, cervical carcinoma (HeLa) and colon adenocarcinoma CaCo-2 cells, hence demonstrating high specificity. Flow cytometric analysis revealed a remarkably low dissociation constant of S15-APTs-decorated QDs to A549 cells (K_d_ = 13.1 ± 1.6 nM). Through the systematic application of a series of established inhibitors of known mechanisms of endocytosis, we show that the uptake of S15-APTs proceeds via a classical clathrin-dependent receptor-mediated endocytosis. This cancer cell-selective mode of entry could possibly be used in the future to evade plasma membrane-localized multidrug resistance efflux pumps, thereby overcoming an important mechanism of cancer multidrug resistance.

## INTRODUCTION

Lung cancer is one of the most common malignancies in the world with 1.6 million new cases diagnosed annually, resulting in 1.4 million deaths [1]. Non-small cell lung cancer (NSCLC) is the dominant class of lung cancer, which represents ~85% of lung cancer cases [2]. Lung cancer is the most lethal malignancy for both men and women. In this respect, each year more cancer patients succumb to lung cancer than to colon, breast, and prostate cancers combined. Current chemotherapy treatment results in low response rates, which emphasizes the burning necessity to develop more effective therapeutic regimens. The main drawbacks of chemotherapeutic treatments are the side effects causing untoward toxicity to healthy tissues and organ dysfunction, where cells are continuously dividing (e.g. bone marrow and gastrointestinal tract). This results in myelosuppression and mucosal epithelium dysfunction, thereby enhancing patient vulnerability to life-threatening pathogenic infections [3]. Another major hindrance towards curative cancer treatment is the frequent emergence of multidrug resistance (MDR), predominantly mediated by efflux transporters of the ATP-binding cassette (ABC) superfamily including P-glycoprotein (P-gp). The latter expels numerous structurally and functionally distinct chemotherapeutics from cancer cells thereby abolishing their cytotoxic activity [4]. The emergence of MDR is believed to result in the failure of anticancer drug treatment in a spectrum of human malignancies, metastatic cancers in particular [5]. A well-studied route to overcome this major hindrance to curative chemotherapy is by achieving internalization of actively targeted nanoparticles (NPs) via selective endocytosis, thereby evading MDR efflux pumps, such as P-gp, which reside in the plasma membrane [6, 7]. Selectively targeted delivery into cancer cells via endocytosis is expected to enable the use of minimal drug doses with maximal treatment efficacy, while dramatically reducing the cost of treatment.

For decades, countless efforts have focused on the development of targeted anticancer chemotherapy aimed at reducing toxic side effects caused by the insufficient selectivity of conventional chemotherapeutic drugs. Active targeting of chemotherapeutic drugs towards malignant cells is a widely studied approach which allows high selectivity of the antitumor drugs, thereby reducing drug doses needed for effective treatment, while simultaneously minimizing side effects of conventional chemotherapy [4, 6–8]. Another important targeting mechanism of NP-based drug delivery systems is passive targeting by the enhanced permeation and retention (EPR) effect. Small (10–200 nm), long-circulating NPs are able to penetrate solid tumors via their leaky blood vessels, thereby remaining within the tumors due to the lack of lymphatic drainage [7, 9].

Nucleic acid APTs are a novel class of molecular probes and are currently attracting a great interest as novel small molecules which can be easily synthesized and directed against any desired target. APTs are short single-stranded DNA or RNA (ssDNA or ssRNA) molecules selected using the technique of systematic evolution of ligands by exponential enrichment (SELEX) [10]. By folding into distinct tertiary structures, APTs can recognize a wide array of targets with high affinity and specificity [10]. In essence, APTs can be prepared against any desirable target and furthermore any cancer type, e.g. S15-APT is an 85 bases long ssDNA selected for specific binding to NSCLC [11]. Facile and low-cost synthesis *in vitro*, along with reduced immunogenicity *in vivo* and rapid tissue penetration, render APT advantageous over other specific ligands, such as antibodies, as drug targeting molecules [10, 11]. Owing to the high potential of APTs, they have been extensively studied in various aspects as targeting agents for various biomedical applications, including cancer diagnosis, antitumor therapy, biomarker identification, and active targeting ligands for advanced drug delivery systems [12–17].

Established endocytosis pathways can be classified into four main routes of internalization: a) clathrin-mediated endocytosis (also known as receptor-mediated endocytosis), b) caveolae-mediated endocytosis, c) macro-pinocytosis, and d) phagocytosis. Receptor-mediated endocytosis (RME) involves a more rapid means of internalization compared to the other internalization mechanisms (i.e. caveolae-mediated endocytosis, macropinocytosis, and phagocytosis) [18]. By the use of receptor-dependent or receptor-independent endocytic pathways, the intracellular trafficking can also be controlled. Detailed knowledge of endocytosis pathways is invaluable, as this information can be translated towards the construction of NPs that can be targeted to specific intracellular compartments, thereby controlling their breakdown, payload release mechanism, and drug target destination [19, 20].

In the current paper, we studied the selectivity of an aptamer (S15-APT) as a potential active targeting ligand against NSCLC, using QDs decorated with this aptamer (S15-APT QDs). We specifically characterized the mode of entry of this aptamer and aptamer-decorated NPs into these tumor cells. We found that the selective internalization of these S15-APT QDs by human NSCLC cells occurs via classical clathrin-dependent, receptor-mediated endocytosis. This finding could be harnessed for the development of actively targeted drug delivery and diagnostic systems, based on S15 APT-decorated NPs, which may selectively enter NSCLC via receptor-mediated endocytosis and thereby bypass MDR efflux transporters.

## RESULTS

### Binding affinity and selectivity to human A549 NSCLC cells

**Figure 1:**
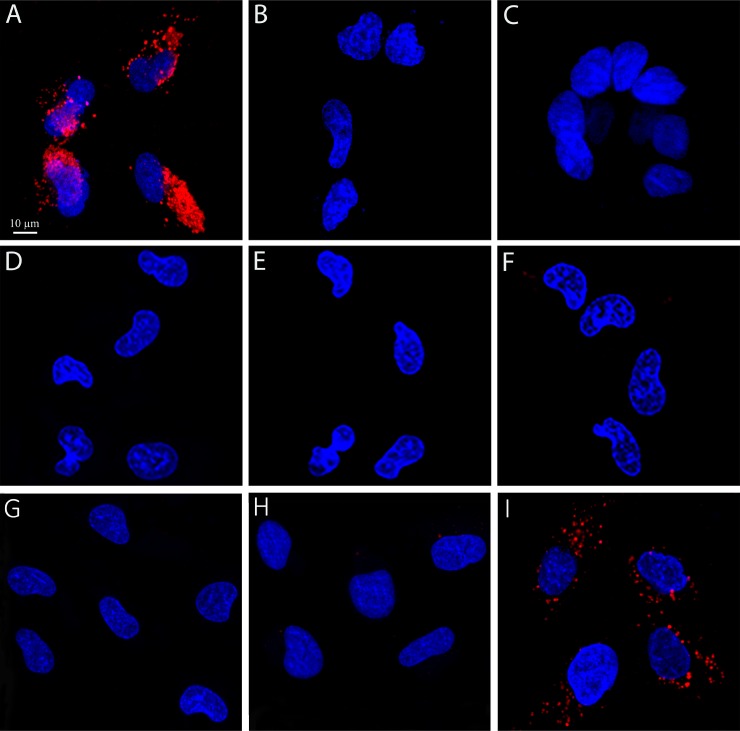
Selective internalization of S15 APTs into human non-small cell lung A549 target cells Cells were incubated with 50 nM S15-APT QDs for 2 h. Nuclei were stained with 2 μg/ml Hoechst 33342. Fluorescence confocal microscopy was performed using an inverted confocal microscope (Zeiss LSM 710) at ×630 magnification. (**A**) Human A549 non-small cell lung carcinoma cells; (**B**) Normal human bronchial epithelial BEAS2B cells which served as normal non-target cells, (**C**) Human colon adenocarcinoma CaCo-2 cells; (**D**) Human cervical carcinoma HeLa cells; (**E**) A549 cells were incubated with 50 nM S15-APT QDs along with 5 μM free APT; (**F**) A549 cells were incubated with 50 nM random sequence APT-QDs; (**G**) A549 cells incubated with no S15-APT QDs; H) A549 cells incubated with 50 nM Qdot^®^ 655; (**I**) ABCG2-overexpressing MDR subline A549/K1.5 cells incubated with 50 nM S15-APT QDs; the red fluorescence channel was defined between 10-100 for all presented images.

The internalization of S15 APT-decorated QDs was explored by confocal laser microscopy in human A549 NSCLC cells and compared to normal human bronchial epithelial BEAS2B, cervical carcinoma (HeLa) and colon adenocarcinoma cells (CaCo-2) (Figure 1). Following an incubation with 50 nM S15-APT QDs for 2 h at 37° C, A549 cells displayed a remarkable internalization of the red fluorescent S15-APT QDs as evidenced by the intense red fluorescent intracellular vesicles that appeared as possible endosomes (Figure 1A). Flow cytometric analysis revealed a saturation S15-APT QDs fluorescence curve, indicating binding to A549 cells. The binding of the S15-APT-decorated QDs to their putative cell membrane target exhibited a very low dissociation constant (K_d_ = 13.1 ± 1.6 [nM]) (Figure 3A), indicating a very high binding affinity. When competitive binding conditions were employed using a 100-fold excess of free S15-APTs, a complete ablation of S15-APTs binding to A549 cells was observed, further establishing that the S15-APT moiety mediates binding to target A549 cells (Figure 3B and C). In contrast, neither normal human bronchial epithelial BEAS2B cells, nor HeLa or CaCo-2 cells showed any cellular fluorescence (Figure 1B, 1C, and 1D, respectively). The selective internalization of the S15-APT QDs into A549 cells was further tested upon incubation with a 100-fold molar excess of the free S15-APT (Figure 1E). This competition with excess free APT completely abolished the internalization of the S15-APT QDs into A549 cells. Moreover, A549 cells incubated with random sequence APT-decorated QDs failed to take up these red fluorescent NPs, further supporting the specificity of S15-APT-QDs to target A549 cells (Figure 1F). A negative control, consisting of A549 cells only, is shown in Figure 1G. Incubation of A549 cells with free QDs^®^ 655 failed to show any cellular fluorescence, further corroborating the indispensable role of the S15 aptamer for the internalization of S15-APT-QDs into A549 cells (Figure 1H). Incubation of HeLa, BEAS2B and CaCo2 cells with free QDs^®^ 655 revealed no cellular fluorescence (results not shown), demonstrating that in the absence of the S15-APT component, free QDs^®^ 655 are unable to undergo internalization. To demonstrate the ability of S15-APT QDs to undergo internalization into cognate MDR cells, ABCG2-overexpressing MDR A549/K1.5 cells were tested for S15-APT QDs internalization. A clear internalization of S15-APT QDs was observed, suggesting the future ability of such S15-APT QDs to evade extrusion by plasma membrane MDR efflux pumps (Figure 1I). These findings warrant future studies with cytotoxic drug-loaded APT-decorated nanoparticles, to evaluate the possible overcoming of cancer MDR.

**Figure 2:**
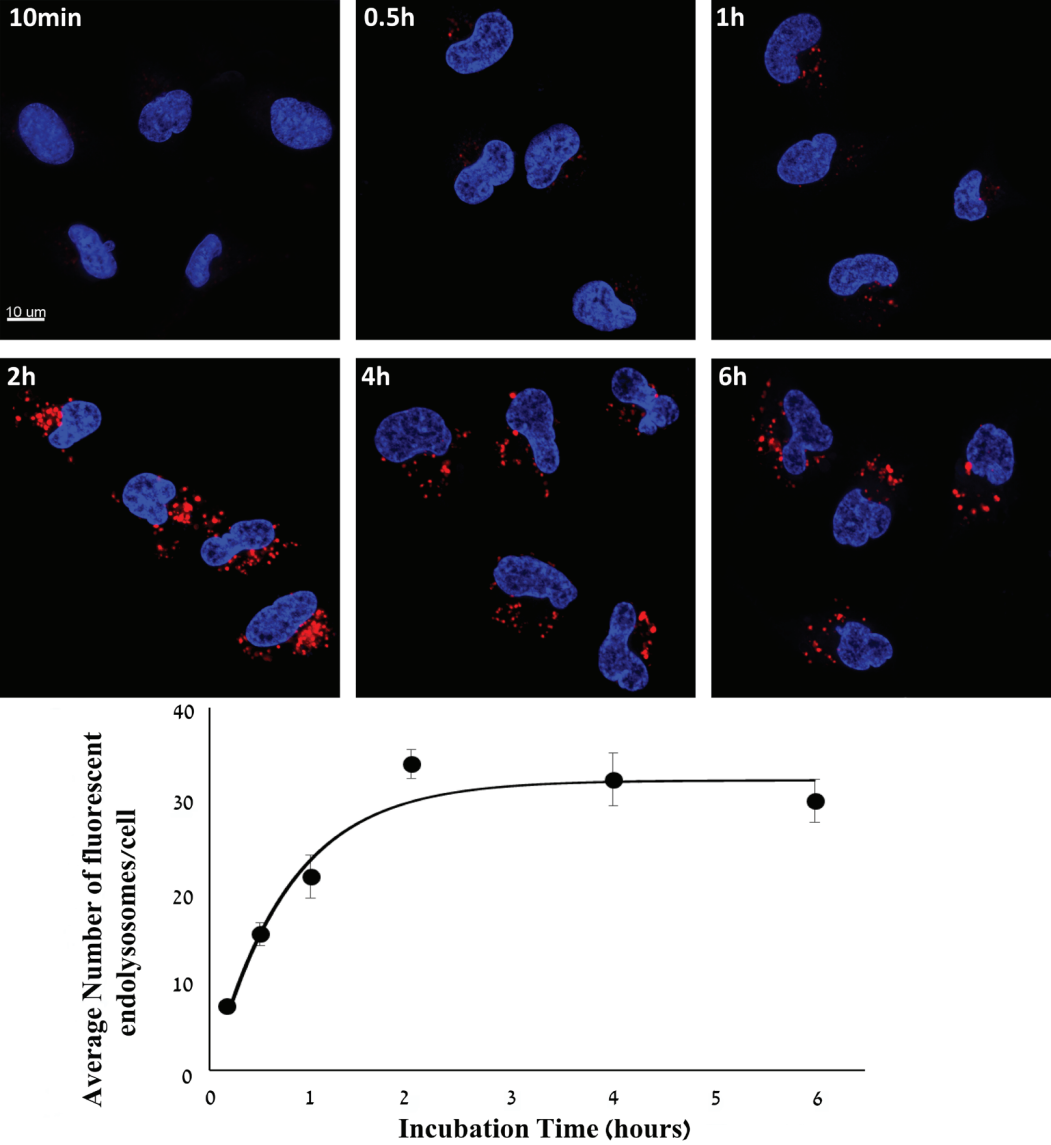
Kinetic study of cellular internalization of S15-APT QDs into A549 cells A549 cells were incubated with 50 nM S15-APT QDs for 10 min, 0.5 h, 1 h, 2 h, 4 h, and 6 h at 37° C. Nuclei were stained with 2 μg/ml Hoechst 33342. Fluorescence confocal microscopy was performed using an inverted confocal microscope (Zeiss LSM 710) at ×630 magnification. Quantification of the average number of endolysosomes per cell was determined using Imaris Software. The red fluorescence channel was defined between 10-100 for all presented images. Fitting the dependence of the average number of fluoresent endolysosomes/cell (Y(t)) to the incubation time (t) was performed by nonlinear curve fitting (Eq. 1).

**Figure 3:**
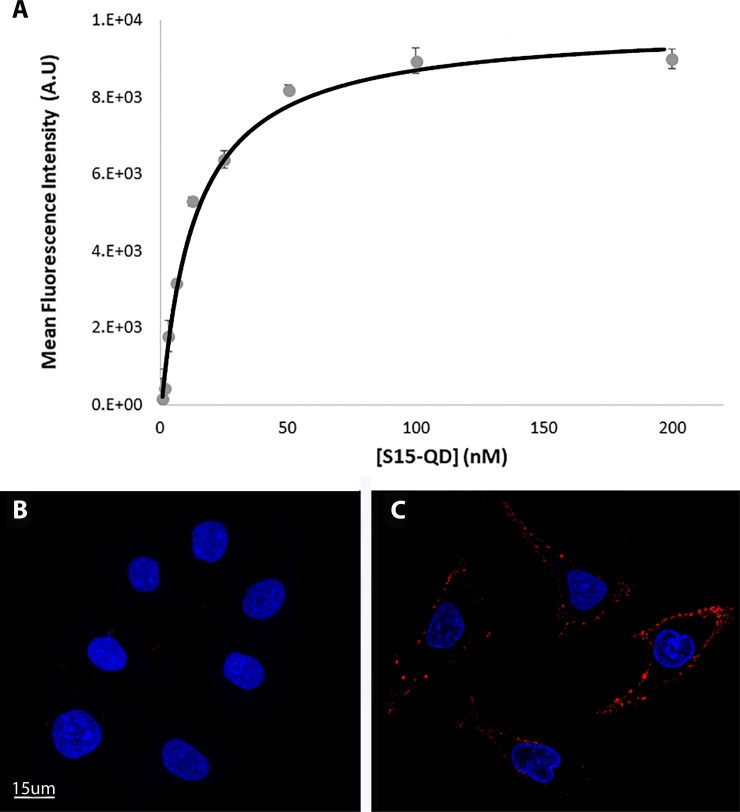
Determination of the dissociation constant of S15-APT from A549 target cells and demonstration of selective binding of S15-APTs to A549 cells (**A**) The equilibrium dissociation constant (K_d_) of the S15-APT-cell interaction was evaluated by flow cytometry. The K_d_ was obtained by fitting the results of mean red fluorescence intensity of specific binding vs. the concentration of the aptamers to a Langmuir model equation (Eq. 2). A549 cells were incubated for 50 min on ice (to prevent internalization) with increasing concentrations of S15-APT QDs from 0.78 to 200 nM. (**B**) A549 cells were pre-incubated with a 100-fold molar excess of free APT for 15 min on ice, followed by incubation with 50 nM S15-APT QDs along with 5 μM free APT on ice; (**C**) A549 cells were incubated with 50 nM S15-APT QDs for 50 min on ice; Nuclei were stained with 2 μg/ml Hoechst 33342. Fluorescence confocal microscopy was performed using an inverted confocal microscope (Zeiss LSM 710) at ×630 magnification. The red fluorescence channel was defined between 10-100 for all presented images.

### Kinetics of cellular internalization of S15-APT QDs into A549 cells

To determine the time-course of internalization of S15-APT QDs into A549 cells, the average number of fluorescent endolysosomes per cell was determined at various time points. After 10 min, almost no red fluorescence was detected, and the average number of fluorescent endolysosomes was 7.0 ± 0.5 per cell. After 0.5 h, 1 h, 2 h, 4 h and 6 h of incubation, the average numbers of fluorescent endolysosomes were: 15.0 ± 1.3, 21.3 ± 2.3, 33.7 ± 1.6, 32.0 ± 2.9, and 29.6 ± 2.3, respectively. Two hours of incubation resulted in a significantly higher uptake of S15-APT QDs than after 1 h (Figure 2). The internalization rate constant K_i_ was calculated from the kinetic study of cellular internalization by nonlinear curve fitting. The T ½ of S15-APT QDs internalization derived from K_i_ was 0.53 ± 0.16 h. The Y_max_ obtained was 31.8 ± 4.7 fluoresent endolysosomes/cell.

### Characterization of the mode of S15-APT uptake into A549 cells

**Figure 4:**
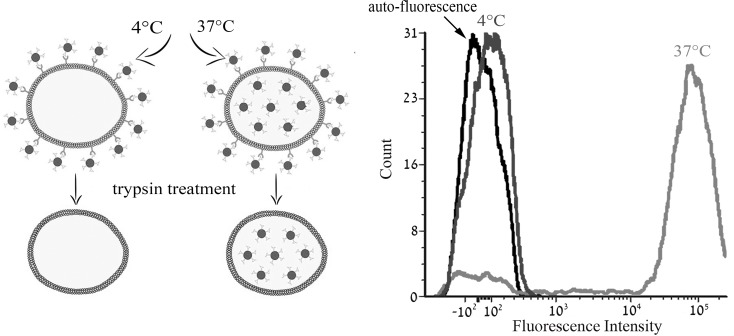
Characterization of the active transport of S15-APT The impact of temperature on cellular accumulation of S15-APT QDs (100 nM) in A549 cells was studied at 4° C vs. 37° C using flow cytometry. A549 cells were incubated for 2 h in growth medium in the absence of S15-APT QDs or in the presence of 100 nM S15-APT QDs at 4° C, or at 37° C. Trypsin treatment was applied to remove the putative target cell surface protein to which S15-APT QDs presumably bind. Cellular fluorescence was determined using flow cytometry. Left panel: schematic diagram of the experimental principle; Right panel: Mean fluorescence intensity.

To determine the mode of entry, we first examined the possible temperature-dependence of S15-APT QDs uptake into A549 cells. Cells were pre-incubated at 4° C, a temperature at which active internalization is disabled, and at 37° C, the physiological temperature at which active uptake proceeds. In each case, the mean cellular fluorescence intensity (M.F.I) was determined by flow cytometry [21]. Following a 2 h incubation with S15-APT QDs, cells were detached by trypsinization for 15 min, which also facilitated the removal of any surface-protein-bound S15-APT QDs. When internalization was studied at 4° C, and any post-incubation surface-protein-bound APT was removed, the fluorescence signal was completely abolished; the M.F.I at 4° C was equivalent to the auto-fluorescence level of unstained A549 cells, indicating that no internalization occurred. In contrast, these S15-APT QDs were readily internalized at 37° C, as evidenced by the >1000-fold increase in cellular fluorescence over the auto-fluorescence level (Figure 4). These findings suggest that the mode of entry is a highly temperature-dependent process which is characteristic of an active uptake process.

**Figure 5:**
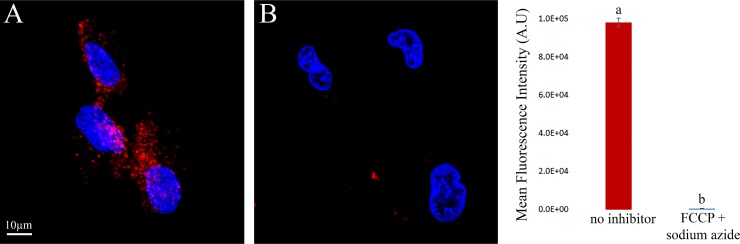
Impact of metabolic energy deprivation on S15-APT internalization in A549 cells Left: (**A**) Control A549 cells incubated with S15-APT QDs (100 nM) for 1 h in inhibitor-free growth medium; (**B**) A549 cells were pre-incubated for 30 min with 5 mM sodium azide and incubated for 1 h with 100 nM S15-APT QDs, along with 5 (μg/ml) FCCP and 5 mM sodium azide, which disrupt mitochondrial ATP synthesis; Right: Mean fluorescence values of panels A and B were determined using IMARIS software. Nuclei were stained with 2 μg/ml Hoechst 33342. Fluorescence microscopy was performed using an inverted confocal microscope (Zeiss LSM 710) at ×630 magnification. The red fluorescence channel was defined between 10–100 for all presented images.

To provide direct evidence that the internalization of S15-APTs is an energy-dependent process, cells were treated with a combination of 5 μg/ml FCCP and 5 mM sodium azide, established metabolic energy inhibitors which abolish mitochondrial ATP synthesis and hence lead to depletion of intracellular ATP stores by inhibiting oxidative phosphorylation and mitochondrial electron transport, respectively [22]. In contrast to the intense red fluorescence signal observed in inhibitor-free control cells due to the internalization of S15-APT QDs, no red fluorescence signal was observed in cells simultaneously treated with these metabolic energy inhibitors, FCCP and sodium azide (Figure 5A and 5B, respectively). These results lend further support that the mechanism of uptake is indeed energy-dependent.

### Clathrin-mediated endocytosis is the mechanism underlying S15-APTs uptake into A549 cells

**Figure 6:**
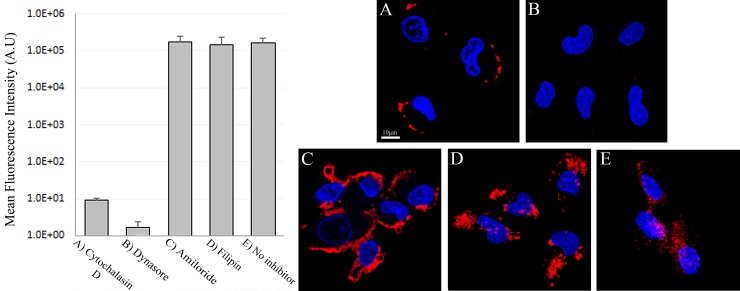
Disruption of clathrin-mediated endocytosis of S15-APTs with different inhibitors Right: A549 cells were pre-incubated with: (**A**) 5 μM cytochalasin D for 30 min to block endocytosis of S15-APT QDs; (**B**) 80 μM Dynasore for 30 min; (**C**) 1 mM Amiloride for 10 min; (**D**) 1 μg/ml Filipin for 30 min; and (**E**) drug-free medium; followed by a further incubation of 2 h with 100 nM S15-APT QDs. Nuclei were stained with Hoechst 33342. Fluorescence microscopy was performed using an inverted confocal microscope (Zeiss LSM 710) at ×630 magnification. Left: Mean fluorescence intensity (M.F.I) values of S15-APT QDs in A549 cells incubated with different inhibitors were determined with IMARIS software for analysis of image data. The red fluorescence channel was defined between 10-100 for all presented images.

**Figure 7:**
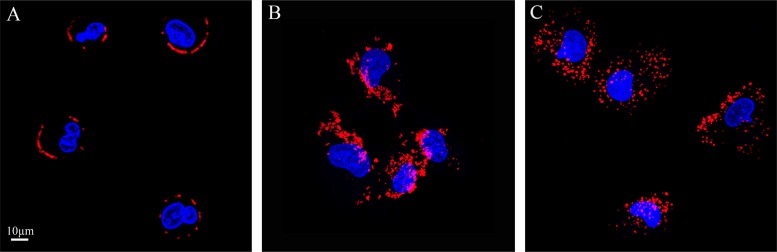
Pitstop-2 confirms that the predominant mechanism of S15-APT internalization is clathrin-mediated endocytosis (**A**) A549 cells were pre-incubated for 10 min with 25 μM Pitstop-2, an established CME inhibitor, followed by incubation for 40 min with 25 μM Pitstop-2 and 100 nM S15-APT QDs; (**B**) A549 cells were pre-incubated for 10 min with 25 μM Pitstop-2 negative control (which does not inhibit endocytosis) and incubated for 40 min with 25 μM Pitstop-2 negative control along with 100 nM S15-APT QDs; (**C**) A549 cells were pre-incubated for 10 min with 25 μM Pitstop-2, then incubated for 50 min in growth medium containing 10% FCS (the medium was refreshed once) to restore the ability of cells to undergo CME. Cells were then incubated with 100 nM S15-APT QDs for 40 min. Nuclei were stained with Hoechst 33342. Fluorescence microscopy was performed using an inverted confocal microscope (Zeiss LSM 710) at ×630 magnification. The red fluorescence channel was defined between 10-100 for all presented images.

**Figure 8:**
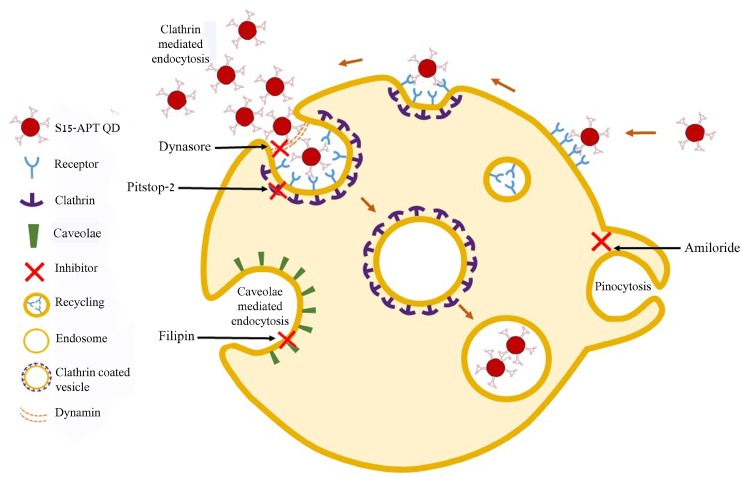
Summary scheme of endocytosis pathways and their specific established inhibitors

To determine whether or not the uptake of S15-APTs proceeds via endocytosis, we first assessed the involvement of the actin cytoskeleton, which has been implicated in regulating endocytosis pathways by trafficking endosomes along the cytoskeleton. A549 cells were pretreated with cytochalasin D, an established actin polymerization inhibitor, which disrupts endocytosis. A549 cells were studied for S15-APT QDs uptake by confocal laser microscopy (Figure 6). Cytochalasin D-treated cells showed a marked decrease in the uptake of S15-APT QDs, consistent with an endocytic uptake (Figure 6A). We further investigated the specific pathway responsible for the putative receptor-mediated endocytosis. For this purpose, A549 cells were pretreated with various established receptor-mediated endocytosis inhibitors (Figure 8 and Table 1) and then studied for S15-APT QDs uptake by confocal laser microscopy (Figure 6B–6D and Figure 7). We found that neither pretreatment with Amiloride (Figure 6C), an inhibitor of pinocytosis, nor with Filipin (Figure 6D), an inhibitor of caveolae-mediated endocytosis, inhibited internalization of S15-APT QDs into A549 cells. No endosomal accumulation was observed in Amiloride-treated cells, whereas in Filipin-treated cells, endosomes were clearly observed. Because the intensities of red fluorescence in the presence of these inhibitors of pinocytosis and caveolae-mediated endocytosis, respectively, were similar to that of the untreated control, the possibility that these pathways play a role as possible internalization routes for S15-APTs uptake was ruled out. In contrast, we observed the complete abolishment of the red fluorescence upon pretreatment of cells with Dynasore, an established dynamin inhibitor (Figure 6B). To further explore the possibility of clathrin-mediated endocytosis (CME), we used Pitstop 2, another potent CME inhibitor (Figure 7A). Pitstop 2 competitively inhibits clathrin box ligands from binding to clathrin terminal domain (TD) (Figure 8) [23]. Figure 7A shows no intracellular red fluorescence in A549 cells, indicating that no internalization occurred in cells pretreated with Pitstop 2. It should be noted, however, that the red fluorescent S15-APT-QDs can be observed on the plasma membrane due to the inhibition of clathrin vesicle formation by Pitstop 2. Distinctly different outcomes were observed between the treatments with Dynasore and Pitstop 2 (Figure 6B and Figure 7A, respectively), as no such fluorescence was observed on the plasma membrane of Dynasore-treated cells.

**Table 1:** Perturbation of endocytosis trafficking pathways

Inhibitor	Effect	Mechanism
Amiloride	Inhibition of pinocytosis	Inhibitor of Na^+^/H^+^ exchange [34]
Filipin	Inhibition of caveolae	Sterol-binding agent causing disassembly of caveolae [42]
Dynasore	Inhibition of CME	Inhibitor of the GTPase activity of dynamin [33]
Pitstop 2	Inhibition of CME	Inhibitor of clathrin box ligands from binding to clathrin TD [23]

A549 cells were also pretreated with the Pitstop 2-negative control, a small molecule with a highly similar structure to Pitstop 2, which unlike the latter, does not block CME (Figure 7B), as evidenced by the presence of intracellular red fluorescent vesicles. Importantly, we also demonstrated that Pitstop 2 elicits a reversible inhibition of CME (Figure 7C), demonstrating that the cells are viable, and upon washing the inhibitor away, cells resumed their ability to take up the S15-APTs via CME. Hence, the established CME inhibitor Pitstop 2 also confirmed that the mechanism underlying S15-APT internalization is CME.

## DISCUSSION

The S15-APT holds great potential as an attractive ligand to specifically target NSCLC [11]. Yet, this molecule was hardly investigated and little is known about its characteristics [24]. In the current paper, we studied the selectivity and specificity of S15-APT and investigated the mechanism underlying its internalization by target A549 cells. This approach could be adopted to target and facilitate receptor-mediated endocytosis of S15 APT-decorated NPs harboring an anticancer drug cargo, hence possibly bypassing mechanisms of MDR that are largely based on the overexpression of MDR efflux pumps located in the plasma membrane [25–27].

Using confocal laser microscopy, we initially demonstrated the selective binding of S15-APT QDs to, and internalization into, A549 cells, while confirming that no binding and internalization occur in normal BEAS2B lung, CaCo-2, or HeLa cells (Figure 1A–1D). The finding that S15-APT are taken up into A549 cells, but not into HeLa cells, is in accord with previous studies [11, 24]; it was previously shown that neither squamous carcinoma NSCLC (NCI-H157), nor NSCLC (NCI-H446) and human breast carcinoma (MCF-7) cells exhibited any internalization of these S15-APT [11]. To confirm that the targeting properties are specifically attributable to the S15-APT, we undertook a competitive inhibition experiment with excess free S15-APTs (that are not conjugated to QDs), which completely abolished the binding to, and internalization of the S15-APT QDs into A549 cells (Figures 1E, 3B, and 3C). Furthermore, A549 cells failed to take up QDs decorated with an 85 bases random oligonucleotide sequence, thus further confirming that the specific sequence of S15-QDs mediates the selective binding and internalization into A549 cells (Figure 1F). To further confirm that the targeting properties of S15-QDs are specifically attributable to the S15-APT, we also incubated A549 cells with free QDs^®^ 655. No red fluorescence was observed, confirming that the binding and internalization of S15-APT QDs into A549 cells are attributable to the S15-APTs component (Figure 1H). We next explored the potential of S15-APT QDs to undergo internalization into MDR cells; we hence tested cognate MDR A549/K1.5 cells, with ABCG2-overexpression [28]. Internalization of S15-APT QDs was observed, demonstrating the potential ability of S15-APT QDs to evade plasma membrane MDR efflux pumps (Figure 1I). A time-course study of S15-APT QDs endocytosis into A549 cells displayed a gradual increase in cellular red fluorescence while attaining a maximal internalization, as reflected in Y_max_ value at 2 h of incubation (Figure 2).

To further corroborate these selective binding results, the affinity of S15-APT QDs to target cells was determined using flow cytometry. S15-APT QDs displayed a striking binding to the plasma membrane of A549 cells with a very low dissociation constant: K_d_ = 13.1 ± 1.6 nM (Figure 3). To verify that this high-affinity binding to A549 cells is attributable to the S15-APT component, competitive binding with a 100-fold molar excess of free S15-APTs was studied using confocal fluorescence microscopy. Indeed, a complete abolishment of aptamer binding to A549 cells was observed, whereas in the absence of competition, a clear red fluorescence on the cell membrane was observed (Figure 3B and 3C, respectively). We therefore concluded that the high-affinity binding and selectivity of S15-APT to A549 cells could possibly serve as an essential feature for the future utilization of this aptamer for targeted delivery of NPs harboring anticancer drug cargo towards NSCLC. Moreover, the K_d_ obtained here is in general agreement with the value observed during the selection of S15-APT (K_d_ = 56.8 ± 3.2 nM) [11]. Some differences in the obtained equilibrium dissociation constant (K_d_) may be due to the incubation of S15-APT QDs with monolayer A549 cells, while in the paper by Zhao *et al*., [11], trypsin-detached A549 cells in suspension were treated with FITC-conjugated S15-APTs. Clearly, the suspension state of A549 cells is not the physiological growth condition of these cells that are naturally adherent monolayer epithelial cells. In the cell suspension state, there can be a distinctly altered regulation of gene expression, possibly causing changes in receptor expression on the cell surface [29]. Another possible explanation for the stronger binding constant in our present study could be the fact that several APTs are conjugated to each QD, and may therefore simultaneously bind to multiple vicinal target receptor proteins, thereby enhancing the binding constant [30, 31]. In contrast, in the paper by Zhao *et al*., [11], a single fluorophore-conjugated APT was used. These findings indicate that the uptake mechanism of S15-APT involves a high-affinity receptor, which is saturated at high nanomolar concentrations. These results are consistent with the finding that the putative target receptor molecule of S15-APT is most likely a plasma membrane protein [11].

To delineate the mode of internalization of S15-APTs by target A549 cells, we used confocal fluorescence microscopy and flow cytometry. To determine whether or not active uptake of S15-APT QDs is effective in A549 cells, we determined the impact of metabolic energy inhibitors and the temperature dependence of S15-APT QDs uptake into A549 cells after trypsin treatment (Figure 4 and Figure 5) [21]. Indeed, the mode of entry was highly temperature-dependent, confirming an active uptake process. Moreover, co-treatment with the metabolic energy inhibitors, FCCP and sodium azide abolished S15-APT uptake, confirming that the uptake mechanism was energy-dependent [22].

To confirm that the uptake of S15-APTs proceeds via endocytosis, we first assessed the involvement of the actin cytoskeleton. The significant decrease in the uptake of S15-APT QDs in cytochalasin D-treated cells was consistent with an endocytosis-based uptake. Moreover, it can be observed that the S15-APT QDs are bound to the cell surface without being able to internalize due to the disruption of actin cytoskeleton fibers (Figure 6A). Since A549 cells are attached monolayer cells, the fluorescence of S15-APT QDs is visible only on one side of each cell - most likely the upper part of the monolayer cell that was exposed to the growth medium and presumably not in the plasma membrane segment that was adherent to the substratum. We hence explored the exact mode of endocytosis by applying established inhibitors, to perturb uptake and subcellular trafficking processes (Figure 8). NPs that undergo internalization through CME are destined for accumulation in lysosomes. Whereas, NPs internalized via a caveolin-mediated process are not [32]. The GTPase dynamin is required for clathrin- and caveolae-mediated endocytosis, as well as some clathrin- and caveolae-independent pathways [33]. We demonstrated that pretreatment with Dynasore, a potent inhibitor of dynamin, leads to a marked decrease in S15-APT QDs uptake into A549 cells (Figure 6B). We hence identified an actin-dependent, and dynamin-dependent pathway for the internalization of S15-APTs into A549 cells. To further pinpoint the specific mechanism of internalization, cells were treated with Amiloride, an inhibitor of macropinocytosis, or with Filipin, an inhibitor of caveolae-mediated uptake. Both inhibitors did not exert any inhibitory effect on the uptake of S15-APT QDs (Figure 6C and 6D, respectively). Amiloride inhibits Na^+^/H^+^ exchange, therefore exerting a major deleterious effect on actin, which is greatly impaired by a decrease in cytosolic pH. This accounts for the inhibition of macropinocytosis and also explains why the S15-APT QDs are not trafficked to the lysosomes, although their entry into the cell remains unperturbed [34]. Pitstop 2, which competitively inhibits the binding of clathrin-ligands, was complemented with dynamin inhibition to achieve a more specific means of CME perturbation (Figure 7) [23]. The observed differences between Dynasore and Pitstop 2 (Figure 6B and Figure 7A) can be explained as follows: when dynamin was inhibited by Dynasore, the budding vesicles were unable to undergo scission, and the S15-APT QDs were washed out from the opened vesicle neck. In contrast, when eliminating the binding of the clathrin box ligands with Pitstop 2, the pits were able to close, but not to detach from the cell membrane, which is clearly observed as a red fluorescent halo under the cell membrane. Had they been on the exterior of the cell membrane, they would have been washed. Hence, these cumulative findings reveal that S15-APT QDs are taken up via a classical CME process.

While achieving all the benefits of targeted delivery, receptor-mediated endocytosis also results in a high degree of biological efficacy [35]. Each APT may possess a distinct mode of internalization e.g. the AS1411-APT is a well-studied APT reported to enter cancer cells via macropinocytosis [15]. In contrast, S15-APTs enter A549 cells via receptor-mediated endocytosis characterized by a significantly higher uptake capacity than macropinocytosis [35, 36]. Another APT that was found to enter cells through CME was suggested to do so after the authors employed sucrose as an inhibitor of clathrin by a hypertonic shock, without using the above compelling inhibitors [14, 19]. Yet another APT conjugated to Ag NPs was claimed to be taken up through caveolae-mediated endocytosis into human neuroblastoma SK-NSH cells; however, this suggestion was not conclusive [37]. Considering that the specific mode of uptake is of great importance for drug delivery, a few efforts have been made to study the cellular uptake mechanism of APTs and the influence on their biological internalization efficacy. By targeting NPs to specific surface receptor protein targets that induce uptake via a classical CME pathway, it is possible to reduce drug doses needed for effective treatment, while simultaneously minimizing the side effects of chemotherapy. Moreover, uptake by CME would facilitate the increase in cellular NPs uptake and should accelerate intracellular trafficking. Drug-loaded nanovehicles often need to be broken down intracellularly to release their drug cargo, hence uptake via CME, which is usually followed by lysosomal coalescence and enzymatic degradation of the NPs, is a highly effective modality to induce therapeutic drug release. Another important potential advantage of CME is the bypass of MDR efflux pumps, an important tactic to evade these potent ATP-driven pumps, thereby overcoming cancer MDR [7, 38, 39].

Taken collectively, these novel findings have important implications for the design of drug delivery NPs targeted by APTs, particularly in the case of S15-APT. Studies are underway that employ S15-APT to direct NPs encapsulating a chemotherapeutic drug cargo into NSCLC cells, including MDR tumor cells, via CME, thereby potentially evading MDR efflux pumps, which constitute a major hindrance to curative chemotherapeutic drug treatment [5]. Lysosomal degradation of the nanovehicle would release the encapsulated drug cargo, thereby facilitating its diffusion into the cytoplasm of cancer cells to achieve potent cytotoxicity. Moreover, understanding how S15-APTs interact with target A549 cells may pave the way towards the identification of candidate biomarkers, which can then be evaluated for both novel cancer diagnostics, monitoring chemotherapy efficacy and for targeted therapeutic interventions. This may also minimize drug doses, while enhancing the efficacy of the chemotherapeutic drug treatment, and dramatically reducing side effects, and the cost of treatment.

## MATERIALS AND METHODS

### Materials

The S15 aptamer with the following nucleotide sequence: 5′-NH_2_-ACG CTC GGA TGC CAC TAC AGG CTA TCT TAT GGA AAT TTC GTG TAG GGT TTG GTG TGG CGG GGC TAC TCA TGG ACG TGC TGG TGA C-3′ [11] was purchased from BioSpring Gesellschaft für Biotechnologie mbH, (Frankfurt, Germany). Qdot^®^ 655 ITK™ carboxyl quantum dots (QY 87%) were obtained from Life Technologies GmbH, (Darmstadt, Germany). The random sequence APT (with the same length as S15) with the following sequence 5′-NH_2_C6-GCA TTT AGT GAC TCG CGG ATG TCA AGA TTA GAC AAC GCC AGC ATC CGA GCC TCT CTA ACT CGT ACG CAG GTC CGA GGC T was purchased from IDT, (Munich, Germany). Cytochalasin D, Filipin III, Dynasore, Amiloride, carbonyl cyanide 4-(trifluoromethoxy) phenylhydrazone (FCCP), Hoechst 33342, and sodium azide were from Sigma-Aldrich Ltd (Rehovot, Israel). Pitstop^®^ 2 and Pitstop^®^ 2 negative control were purchased from abcam (Tel Aviv, Israel).

### Conjugation of quantum dots

The aptamer S15 was conjugated to carboxyl-terminal QDs as previously described [40] with some modification. In brief, 0.75 nmol S15 were added to 0.1 nmol Qdot^®^ 655 ITK™ carboxyl quantum dots in 10 mM sodium borate buffer pH 7.4 in a total volume of 100 μl. After 5 min incubation at room temperature, 3 μl of 10 mg/ml EDC (1-Ethyl-3-(3-dimethylaminopropyl)-carbodiimide) solution (freshly prepared in 10 mM sodium borate buffer at pH 7.4) was added to the mixture and incubated for 18 h at room temperature. S15-conjugated QDs were purified via centrifugal concentrators (100 kDa MWCO PES, Vivaspin, Sartorius Stedim Biotech GmbH, Germany) in order to remove free aptamer. The purification step was repeated 8 times using 50 mM sodium borate buffer at pH 8.3.

As a negative control, QDs were conjugated with a random oligonucleotide using the same procedure. Conjugation was confirmed using 1.5% agarose gel electrophoresis. The amount of the conjugated aptamer was determined colorimetrically via agarose gel electrophoresis using different concentrations of S15 as a reference. This allowed the determination of the amount of the conjugated aptamer by quantification of the aptamer remaining in solution after EDC coupling. The concentration of aptamer-conjugated QDs was determined via nanoparticle tracking analysis (Malvern NanoSight LM10). The described procedure resulted in NPs conjugated with 5 aptamers per QD.

### Cell culture and drug treatment

Normal human bronchial epithelial BEAS2B cells were generously provided by Prof. Rotem Karni (The Hebrew University, Jerusalem, Israel). Human NSCLC A549, HeLa cervical carcinoma, and BEAS2B cells were maintained in RPMI-1640 medium, supplemented with 10% fetal bovine serum, 2 mM glutamine, 100 μg/ml penicillin and streptomycin (Biological Industries, Beit-Haemek, Israel). CaCo-2 cells were maintained in DMEM medium, supplemented with 20% fetal bovine serum, 2 mM glutamine, 100 μg/ml penicillin and streptomycin (Biological Industries, Beit-Haemek, Israel). Cells were incubated at 37° C in a 5% CO_2_ humidified atmosphere.

### The impact of various inhibitors on cellular internalization of S15-APT QDs

Cells were plated in μ-slides VI 0.1 (Ibidi, Martinsried, Germany) at 50% confluence and incubated for 18 h to allow for cell adherence. Cells were then washed with PBS. To investigate the mechanism of internalization of the S15-APT QDs, A549 cells were pre-incubated at 37° C in the presence of different inhibitors: 5 μM cytochalasin D, 1 μg/ml Filipin and 80 μM Dynasore for 30 min; 1 mM Amiloride, 25 μM Pitstop-2 and 25 μM Pitstop-2 negative control for 10 min [14, 15, 23]. The serum-free medium containing these inhibitors was removed by aspiration and 100 nM S15-APT QDs in serum-free medium were then added to the attached cells followed by incubation for 2 h at 37° C. Then, cells were washed three times with PBS. To achieve nuclear staining prior to fluorescence imaging, cells were incubated with 2 μg/ml of the DNA dye Hoechst 33342 in growth medium for 10 min. Fluorescence confocal microscopy was performed using an inverted confocal microscope (Zeiss LSM 710).

A549 cells were pre-incubated at 37° C with 5 mM sodium azide in serum-free medium for 30 min and then incubated for 1 h with 100 nM S15-APT QDs, along with 5 μg/ml FCCP and 5 mM sodium azide (NaN_3_) in serum-free medium [22]. Thereafter, cells were washed three times with PBS to remove unbound S15-APT QDs. To achieve nuclear staining prior to fluorescence imaging, cells were incubated with 2 μg/ml Hoechst 33342 in growth medium for 10 min. The cellular fluorescence pattern was followed using an inverted confocal microscope (Zeiss LSM 710). Images were analyzed with IMARIS software. Two fluorescence channels were used during all image capturing: (1) Blue for the viable DNA-dye Hoechst 33342; and (2) Red for the viable Qdot^®^ 655.

### Live cell confocal imaging

Selective internalization of S15 APTs: A549, BEAS2B, HeLa and CaCo-2 cells were plated on μ-slides VI 0.1 at 50% confluence and incubated for 18 h to allow cell attachment, then cells were washed with PBS. For internalization studies, the growth medium was replaced by serum-free medium containing 50 nM S15-APT QDs, or 50 nM random sequence APT-QDs as a negative control, and incubated for 2 h at 37° C [13]. The selective internalization of S15-APT QDs into A549 cells was further tested after pre-incubation for 30 min with a 100-fold excess competitor consisting of 5 μM free APT, followed by incubation with 50 nM S15-APT QDs, along with 5 μM free APT. To verify that the selective internalization is based on the S15-APTs, A549 cells were incubated for 2 h with 50 nM free Qdot^®^ 655. Furthermore, to examine the potential of these S15-APTs to undergo internalization into MDR cells, ABCG2-overexpressing A549/K1.5 cells were incubated with 50 nM S15-APT QDs for 2 h [28]. Cells were then washed three times with PBS to remove unbound S15-APT QDs. To achieve nuclear staining prior to fluorescence microscopy, cells were incubated with 2 μg/ml Hoechst 33342 in growth medium for 10 min. Fluorescence was followed using an inverted confocal microscope.

Kinetic study of cellular internalization of S15-APT QDs into A549 cells: A549 cells were plated on μ-slides VI 0.1 (Ibidi, Martinsried, Germany) at 50% confluence and incubated for 18 h to allow cell attachment, then, cells were washed with PBS. The growth medium was replaced by serum-free medium containing 50 nM S15-APT QDs and incubated for various incubation times including 10 min, 30 min, 1 h, 2 h, 4 h, and 6 h at 37° C. Cells were then washed three times with PBS to remove unbound S15-APT QDs. To stain the nuclei prior to fluorescence microscopy, cells were incubated with 2 μg/ml Hoechst 33342 in growth medium for 10 min. Fluorescence microscopy was followed using an inverted confocal microscope. The internalization rate constant Ki was calculated from the kinetic study of cellular internalization by nonlinear curve fitting according to Equation 1 :

Equation 1: Y(t) = Y_0_ + (1-e^-Ki×t^)×Y_max_

Fitting the dependence of the average number of fluoresent endolysosomes/cell (Y(t)) to the incubation time (t) was performed using Origin software. Y_0_ and Y_max_ are the initial and maximal numbers of fluoresent endolysosomes/cell, respectively [41].

Selective binding of S15 APTs to A549 cells: A549 cells were plated on μ-slides VI 0.1 at 50% confluence and incubated for 18 h to allow for cell attachment. Cells were then washed with PBS. The growth medium was replaced by serum-free medium containing 50 nM S15-APT QDs and incubated for 50 min on ice. Selective binding of S15-APTs to A549 cells was further assessed after pre-incubation for 15 min with a 100-fold excess competitor consisting of 5 μM free APTs, followed by continuous 50 min incubation with these 50 nM S15-APT QDs along with 5 μM free APTs. Cells were then washed three times with PBS to remove unbound S15-APT QDs. To stain the nuclei prior to fluorescence imaging, cells were incubated with 2 μg/ml Hoechst 33342 in growth medium for 10 min. Fluorescence was followed using an inverted confocal microscope.

### Flow cytometric analysis

Determination of the binding affinity of S15 APTs to A549 cells: A549 cells (1 × 10^5^) were plated in 24-well plates (*In Vitro* Scientific, CA, USA). Following 18 h of incubation at 37° C, cells were first washed with 1 ml PBS to remove serum remnants. The binding affinity of S15-APTs was determined by incubation with increasing concentrations of S15-APT QDs in serum-free medium on ice for 50 min in the dark, in a total volume of 0.25 ml. Cells were then washed three times with 1 ml PBS to remove unbound S15-APT QDs. The cells were then incubated with 0.5 ml Hank's Balanced Salt Solution (HBSS) containing 0.05% trypsin (Fisher Biotech), 0.53 mM EDTA at 37° C for 2 min. After incubation, 50 μl FCS was added. Cells were then transferred to 2 ml Eppendorf microfuge tubes and centrifuged at 800xg for 3 min at room temperature. The supernatant was aspirated and the cells were suspended in 0.5 ml growth medium. Flow cytometric analysis was performed using an LSR-II flow cytometer (BD Biosciences).

The mean fluorescence intensity (M.F.I) of target cells incubated with S15-APT QDs was used to calculate the specific binding of S15-APT QDs by subtracting the auto-fluorescence intensity of A549 cells. Assuming a Langmuir's binding isotherm, Equation 2 was used to obtain the equilibrium dissociation constant (K_d_) of the aptamer-cell interaction:

Equation 2: Y = B_max_ × X/(K_d_ + X)

Fitting the dependence of fluorescence intensity of specific binding (Y) to the concentration of the aptamers (X) was performed using Origin software. B_max_ is the M.F.I at saturation [12].

Characterization of active internalization: A549 cells (1 × 10^5^) were plated in 24-well plates. Following 18 h of incubation at 37° C, cells were first washed with 1 ml PBS to remove serum remnants. Next, a serum-free medium containing 100 nM S15-APT QDs was added for 1 h and incubated at two different temperatures: 4° C and 37° C. Following incubation, cells were washed three times with 1 ml PBS and then incubated with an HBSS buffer containing 0.05% trypsin, 0.53 mM EDTA at 37° C for 15 min. After incubation, 50 μl FCS were added [21] and the cells were then transferred to 2 ml Eppendorf microfuge tubes and centrifuged at 800×g for 3 min at room temperature. The supernatant was aspirated and the cells were suspended in 0.5 ml growth medium. Flow cytometric analysis was performed using an LSR-II flow cytometer.

### Statistical analysis

Results were obtained from three or more independent experiments performed on separate days. Error bars in all Figures represent standard deviation. The statistical significance (*p* < 0.05) of the differences was evaluated using the unpaired two-sided student's *t*-test.
